# Fermented Wheat Bran Polysaccharides Intervention Alters Rumen Bacterial Community and Promotes Rumen Development and Growth Performance in Lambs

**DOI:** 10.3389/fvets.2022.841406

**Published:** 2022-03-30

**Authors:** Wenwen Wang, Yuan Wang, Zhiwei Cui, Yi Yang, Xiaoping An, Jingwei Qi

**Affiliations:** ^1^College of Animal Science, Inner Mongolia Agricultural University, Hohhot, China; ^2^Inner Mongolia Herbivorous Livestock Feed Engineering and Technology Research Center, Hohhot, China

**Keywords:** fermented wheat bran polysaccharides, growth performance, rumen development, rumen bacteria, lambs

## Abstract

There is growing interest in the utilization of plant polysaccharides for the modulation of the rumen bacterial community and enhancement of growth performance in ruminants. Fermented wheat bran polysaccharides (FWBPs), plant polysaccharides, have been shown to improve the growth performance of lambs, but little is known about their effect on rumen bacteria. The aim of this study was to investigate the effects of FWBPs supplementation to milk replacer (MR) on the growth performance, blood metabolites, weight and morphology of rumen, rumen fermentation, and rumen bacterial community which were investigated in lambs. Twelve 1.5-month-old crossbred lambs (Dorper × Small-tailed Han Sheep) with an initial body weight (BW) of 11.38 ± 0.19 kg were randomly divided into two groups, namely, the control group and FWBPs group. Compared with the control group, the FWBPs group had a higher average daily weight gain and serum total protein concentrations, and a lower feed: gain ratio. A tendency of increase in final BW and carcass BW was also observed. Administration of FWBPs increased the ruminal papillae width and ruminal butyrate proportion and decreased the concentration of ammonia nitrogen and the proportion of isobutyrate and isovalerate. In addition, the epithelial cell thickness had an increased trend in the FWBPs group. High-throughput sequencing data showed that the relative abundance of *Lachnospiraceae_NK3A20_group* and *Solobacterium* was enhanced by FWBP treatment; meanwhile, the relative abundance of *NK4A214_group, Megasphaera*, and *Treponema* showed a tendency to be higher than that of the control group. Furthermore, Spearman's correlation analysis revealed that the relative abundances of *NK4A214_group, Treponema*, and *Lachnospiraceae_NK3A20_group* were positively correlated with butyrate proportion. Collectively, FWBPs supplementation to MR on lambs altered the rumen bacterial community, promoted rumen development, and improved growth performance.

## Introduction

Development of the rumen is critical for efficient fermentation, which can determine the lifelong performance and productivity of ruminants (1). The efficient fermentation of rumen is a complicated process conducted by microbiota, which produces volatile fatty acids (VFAs) (1). VFAs can stimulate the growth of rumen epithelia and papillae and promote rumen development (2). Therefore, rumen microbiota play a crucial role in rumen development. The structure and composition of rumen microbiota in young ruminants are significantly affected by plant polysaccharides, such as beta-glucans, mannans, xylans, pectins, and inulin (3). According to the reported data, supplementation of beta-glucans increased *Fibrobacter* populations and the total VFAs concentration in rumen (4), which would help promote the rumen development of calves (5). It has also been reported that xylans can stimulate the growth of *Prevotella* spp. (*ruminicola, albensis, brevis*, and *bryantii*), the most predominant bacterium in the rumen, and increase the production of VFAs and promote rumen development (6, 7).

Similar to plant polysaccharides, fermented wheat bran polysaccharides (FWBPs) were formed mainly by arabinoxylan, which is manufactured from wheat bran by fungal and bacterial strains (8). Previous research has shown that FWBPs supplementation can modify the rumen fermentation profile, increase feed intake and nutrient digestibility, and thereby enhance the growth performance of lambs (9, 10). Furthermore, the prebiotic effects of FWBPs have been demonstrated in monogastric animals which promoted the proliferation of *Butyrivibrio fibrisolvens, Roseburia, Ruminococcus productus, Ruminococcus obeum, Prevotella*, and *Bifidobacteria* (11). However, there is a lack of clarity regarding the FWBPs' effects on the rumen bacteria of suckling lambs. We hypothesized that the positive effects of FWBPs supplementation to milk replacer (MR) on suckling lambs may be due to the impact on rumen bacterial colonization, rumen fermentation, rumen development, and growth performance. Consequently, the effects of FWBPs supplementation to MR on the growth performance, blood metabolites, weight and morphology of rumen, rumen fermentation, and rumen bacterial community were investigated in suckling lambs.

## Materials and Methods

### Ethics Statement

The current study approval for experimental protocols on animals was provided by the Animal Care and Use Committee of Inner Mongolia Agricultural University [(2020)069]. All experimental protocols on animals, including euthanasia, sample collection, and carcass disposal procedures, were in strict accordance with the requirement of the Ethics Procedures and Guidelines of the People's Republic of China.

### Preparation of FWBPs

FWBPs were prepared from wheat bran by *Bacillus subtilis* (CGMCC No. 1.0892) and *Saccharomyces cerevisiae* (CGMCC No. 2.119) fermentation according to our previously reported study (8). Briefly, the wheat bran was inoculated with 10.4% (v/v) inoculum (final concentration of 1 × 10^8^ CFU/ml) which was prepared by mixing activated *S. cerevisiae* and *B. subtilis* in a 3:7 ratio. Then, distal water was added to obtain a 1:1.16 material-to-water ratio. The fermentation was carried out for 48 h at 35°C, followed by drying fermented wheat bran for 24 h at 40°C. The dried substrate was mashed into powder (1.0 mm), and their extraction was performed with distilled water (16:1, v/w) at 90°C for 35 min and centrifuged at 5,000 g for 15 min. The resultant centrifuged solution was deproteinized three times by an equal volume of Savage solution (chloroform: butyl alcohol in 4:1 ratio) (12). The deproteinized solution was mixed with absolute ethanol at a final concentration of 80%, stirred vigorously, and left overnight at 4°C. The samples were centrifuged at 5,000 g for 15 min, and the precipitate was dissolved, collected, and lyophilized to obtain the FWBPs. According to PMP-HPLC evaluation, the monosaccharide content of FWBPs was xylose, glucose, arabinose, galactose, mannose, and rhamnose with percentage compositions of 35.38, 29.15, 24.29, 5.70, 3.17, and 2.31%, respectively (13).

### Experimental Design, Diets, and Housing

Twelve 1.5-month-old crossbred lambs (Dorper × Small-tailed Han Sheep) with an initial body weight (BW) of 11.38 ± 0.19 kg were randomly divided into two groups. The lambs in the control group (CON) were fed MR, whereas the lambs of the FWBPs group (FWBPs) were provided the same MR-containing FWBPs. The amount of FWBPs was adjusted in direct accordance with 2‰ of the amount of MR. The quantity of MR was supplied based on 2% of the lamb's body weight. Meanwhile, the starter supplement of the CON group was assigned to be fed *ad libitum*, and the feed amounts of the FWBPs groups were according to the intake of the CON group. The starter and MR are provided by Beijing Sanyuan Well-hope Agri-Tech Co., Ltd. (Beijing, China) and Precision Animal Nutrition Research Center (Beijing), respectively. The average pellet size was 10 mm in length and 4 mm in diameter. The nutritional composition of the MR and the starter feed is listed in Table 1.

**Table 1:** The nutrients levels of milk replacer and starter (air-dry basis, %).

**Items**	**Milk replacer**	**Starter**
Nutrient levels^a^		
Metabolizable energy ME (MJ/kg)	11.15	13.14
Dry matter	95.52	88.51
Crude protein	24.69	18.07
Ether extract	17.50	4.54
Ash	5.81	6.27
Neutral detergent fiber	-	20.93
Calcium	1.02	0.74
Phosphorus	0.66	0.72

a *The nutrient levels are measured values except ME. ME of milk replacer and starter were calculated according to feed composition and nutritive values in China 2012 and Feeding Standard of sheep (NY/T 816-2004)*.

Animals were housed in separate pens with facilities for individual feeding and watering in a naturally ventilated barn with windows. Lambs were fed at 8:00, 12:00, and 18:00 h, and water was available *ad libitum*.

### Sampling

All lambs were slaughtered using Halal methods in the local abattoir at the end of the experiment. According to the local abattoir procedure, lambs were fasted 24 h with free access to water. The time lag between slaughtering and ruminal fluid sampling was ~25 min. After slaughter, the rumen, reticulum, omasum, and abomasum were removed and weighted. The rumen contents were individually collected into 2.5 ml sterile polypropylene centrifuge tubes, snapped frozen in liquid nitrogen, and kept at −80°C for 16S ribosomal RNA-based taxonomic analysis. Approximately 0.2 L of ruminal fluid was collected from each lamb and strained through 4 layers of cheesecloth. Ruminal fermentation parameters were measured using a 5 ml filtrate subsample.

Tissue samples, 1.5 by 1.5 cm^2^, were removed from the ventral region of each lamb's rumen. After that, tissue samples were then rinsed with sterile phosphate-buffered saline. Specimens of rumen were fixed in 4% paraformaldehyde for the analysis of histomorphology.

### Animal Performance

All lambs were weighed on days 0, 15, and 28 of the experiment. The average daily gain (ADG), average daily feed (MR and starter) intake, and feed-to-gain ratio (F:G) ratio were calculated using daily measurements of MR and starter intake.

### Blood Metabolites

Before the morning feeding on day 28 of the trial, blood samples from 12 lambs were collected into vacutainer tubes (BD, 5.0 ml, gel and clot activator) through jugular vein puncture. After centrifuging at 3,000 g at 4°C for 15 min, the obtained serum was immediately transported to the laboratory for blood metabolite (glucose, total protein, urea nitrogen, total triglyceride, and total cholesterol) determination. The concentrations of blood metabolites were measured with an Automatic Biochemical Analyzer (NSA-300; Neusoft Medical Systems Co., Ltd., Shenyang, China) using colorimetric methods.

### Histomorphological Analysis

The rumen morphology was assessed using the method described by Yi et al. (14). Briefly, rumen samples were dehydrated in a graded series of ethanol before being embedded in paraffin. Next, the preparation of each sample's cross sections was performed, followed by staining with hematoxylin and eosin, and then sealed with neutral resin. The papillae width, papillae length, muscle layer thickness, and epithelial cell thickness of each rumen were measured using a Motic BA610 microscope equipped with a digital camera (Motic China Group Co., Ltd., China) and Motic DS Assistant Lite morphological analysis software.

### Ruminal Fermentation Parameters

The pH of the ruminal fluid was measured immediately after collection *via* a pH meter (Starter 2100, Ohaus Corp., Parsippany, NY, USA). Ruminal VFA was measured with a gas chromatograph (Clarus 680, PerkinElmer, Inc., Waltham, MA, USA) using an Elite-FFAP column (30 m in length with a 0.25 mm i.d.) as described in Chen et al. (15). The concentration of ammonia nitrogen (NH_3_-N) in the ruminal fluid was determined with the colorimetric method described by Ma et al. (16).

### 16S Ribosomal RNA-Based Taxonomic Analysis

The total DNA from the rumen content samples was extracted using the QIAamp DNA Stool Mini Kit (Qiagen, Hilden, Germany) according to the manufacturer's protocols. 16S rRNA genes were amplified by PCR from the genomic DNA samples using specific primers for the V4 region of the bacterial 16S rRNA. Amplicon libraries for all samples were quantified by a Qubit 2.0 Fluorometer (Thermo Fisher Scientific, Waltham, USA) and sequenced on an NovaSeq 6000 platform by Novogene Bioinformatics Technology Co., Ltd. (Beijing, China) to generate 2 × 250 bp paired-end reads.

The singletons and chimeras were removed, and then tags were clustered into the operational taxonomic unit (OTU) using UPARSE (V 7.0.1001, http://www.drive5.com/uparse/) at 97% similarity (17). Afterward, the representative sequences of the OTUs were annotated using the SSUrRNA database (SILVA138, http://www.arb-silva.de/) (18). The alpha and beta diversity were analyzed by QIIME (19).

Linear discriminant analysis coupled with effect size (LEfSe) was conducted to identify bacterial taxa differentially represented among the groups at various taxonomy levels. An LDA effect size of more than 2.5 was used as threshold for the LEfSe analysis.

### Statistical Analysis

The data were analyzed by SAS 9.2 (SAS Institute, Cary, NC, USA) as a completely randomized design. Individual lambs served as the experimental unit. A non-parametric test was employed to assess the bacterial data, and the Wilcoxon rank-sum test was used for multigroup independent samples. The *t*-test was used to analyze the remaining data. The results were expressed as least square means and SEM. Values *p* < 0.05 were taken to indicate the significance, and 0.05 ≤ *p* < 0.10 was taken as an indication of tendency. The correlations between altered rumen bacteria (genus) and rumen fermentation parameters which were significantly affected by FWBPs treatments were demonstrated by Spearman's correlation analysis.

## Results

### Growth Performance of Lambs

Table 2 displays the effects of FWBPs in MR on the growth performance of lambs. The initial BW of early-weaned lambs was found to be similar between the two groups (*p* > 0.10). Administration of FWBPs increased the ADG and decreased the F:G ratio of lambs than the control group (*p* < 0.05). Additionally, the final BW and carcass BW tended to improve in the FWBPs group (*p* = 0.08).

**Table 2:** Effects of FWBPs on the growth performance of early-weaned lambs.

**Items**	**CON**	**FWBPs**	**SEM**	***p*-value**
Initial BW (kg)	11.35	11.41	0.191	0.89
Final BW (kg)	17.28	19.09	0.512	0.08
ADG (g/d)	214.88	273.93	13.687	0.02
Milk replacer intake (g/d)	220	220	-	-
Starter intake (g/d)	595	595	-	-
F:G ratio	3.84	2.99	0.200	0.02
Carcass BW (kg)	7.08	8.38	0.366	0.08
Slaughter rate %	44.45	46.15	0.912	0.41

*CON, the milk replacer (n = 6); FWBPs, supplemented with 2‰ fermented wheat bran polysaccharides in the milk replacer (n = 6).*

*BW, body weight; ADG, average daily gain; F:G ratio, feed to gain ratio; SEM, standard error of the mean*.

### Blood Metabolites of Lambs

The current study revealed that FWBPs treatment had no significant effect on the lamb's serum glucose, blood urea nitrogen, total triglyceride, or total cholesterol concentrations (*p* > 0.10). However, oral infusion with FWBPs raised serum total protein concentrations, as represented in Table 3 (*p* < 0.05).

**Table 3:** Effect of FWBPs on serum metabolic parameters of early-weaned lambs.

**Items**	**CON**	**FWBPs**	**SEM**	***p*-value**
Glucose (mmol/L)	5.01	5.05	0.170	0.91
Total Protein (g/L)	55.54	61.96	1.667	0.04
Blood urea nitrogen (mmol/L)	4.00	3.55	0.166	0.24
Triglyceride (mmol/L)	0.30	0.23	0.029	0.29
Cholesterol (mmol/L)	1.72	1.60	0.099	0.58

*CON, the milk replacer (n = 6); FWBPs, supplemented with 2‰ fermented wheat bran polysaccharides in the milk replacer (n = 6).*

*SEM, standard error of the mean*.

### Rumen, Reticulum, Omasum, and Abomasum Weight of Lambs

As shown in Table 4, no difference was observed for rumen, reticulum, omasum, and abomasum weights and the relative weight of the whole stomach between the two groups (*p* > 0.10).

**Table 4:** Effects of FWBPs on the rumen, reticulum, omasum, and abomasum weight of early-weaned lambs.

**Items**	**CON**	**FWBPs**	**SEM**	***p*-value**
Rumen weight (g)	324.12	353.96	15.922	0.39
Rumen weight/total stomach weight %	63.15	61.63	0.898	0.43
Reticulum weight (g)	52.37	53.93	2.582	0.78
Reticulum weight/total stomach weight %	10.25	9.40	0.377	0.29
Omasum weight (g)	33.57	43.85	4.161	0.24
Omasum weight/total stomach weight %	6.53	7.43	0.402	0.30
Abomasum weight (g)	103.95	124.39	9.105	0.29
Abomasum weight/total stomach weight %	20.06	21.54	0.893	0.45

*CON, the milk replacer (n = 6); FWBPs, supplemented with 2‰ fermented wheat bran polysaccharides in the milk replacer (n = 6).*

*SEM, standard error of the mean*.

### Rumen Morphology of Lambs

Rumen morphology characteristics are summarized in Table 5. The width of ruminal papillae increased after an oral infusion of FWBPs (*p* < 0.05). However, no considerable differences were found in ruminal papillae length or muscle layer thickness between the control and FWBP groups (*p* > 0.10). In addition, the epithelial cell thickness had an increased trend in the FWBPs group (*p* = 0.08).

**Table 5:** Effects of FWBPs on the ruminal morphology of early-weaned lambs.

**Items**	**CON**	**FWBPs**	**SEM**	***p*-value**
Papillae length	1579.3	1593.5	123.294	0.96
Papillae width	379.79	479.45	24.676	0.04
Muscle layer thickness	1479.3	1643.5	166.842	0.66
Epithelial cell thickness	144.29	187.81	12.543	0.08

*CON, the milk replacer (n = 6); FWBPs, supplemented with 2‰ fermented wheat bran polysaccharides in the milk replacer (n = 6).*

*SEM, standard error of the mean*.

### Rumen Fermentation Parameters of Lambs

As shown in Table 6, ruminal pH ranged from 5.65 to 6.12 and was not affected by FWBPs treatment (*p* > 0.1). The concentration of NH_3_-N was decreased (*p* < 0.05) in response to the infusion of FWBPs. Oral infusion with FWBPs increased (*p* = 0.05) the butyrate proportion, whereas it decreased (*p* < 0.05) the isobutyrate and isovalerate proportion. The FWBPs treatment had no effect on the other ruminal fermentation parameters (*p* > 0.1).

**Table 6:** Effects of FWBPs on the ruminal fermentation parameters of early weaned lambs.

**Items**	**CON**	**FWBPs**	**SEM**	***p*-value**
pH	6.12	5.65	0.191	0.25
NH_3_-N (mg/dL)	11.41	5.24	1.353	<0.01
Total VFA (mmol/L)	9.45	12.23	1.375	0.38
**Individual VFA (% total VFA)**				
Acetate	40.15	39.33	0.493	0.45
Propionate	45.38	46.44	1.224	0.70
Butyrate	6.03	9.19	0.840	0.05
Isobutyrate	1.76	0.70	0.228	<0.01
Valerate	3.38	3.69	0.437	0.75
Isovalerate	1.80	0.65	0.274	0.02
Acetate: propionate	0.89	0.85	0.027	0.57

*CON, the milk replacer (n = 6); FWBPs, supplemented with 2‰ fermented wheat bran polysaccharides in the milk replacer (n = 6).*

*NH3-N, ammonia nitrogen; Total VFA, total volatile fatty acid; SEM, standard error of the mean*.

### Rumen Bacteria of Lambs

**Figure 1:**
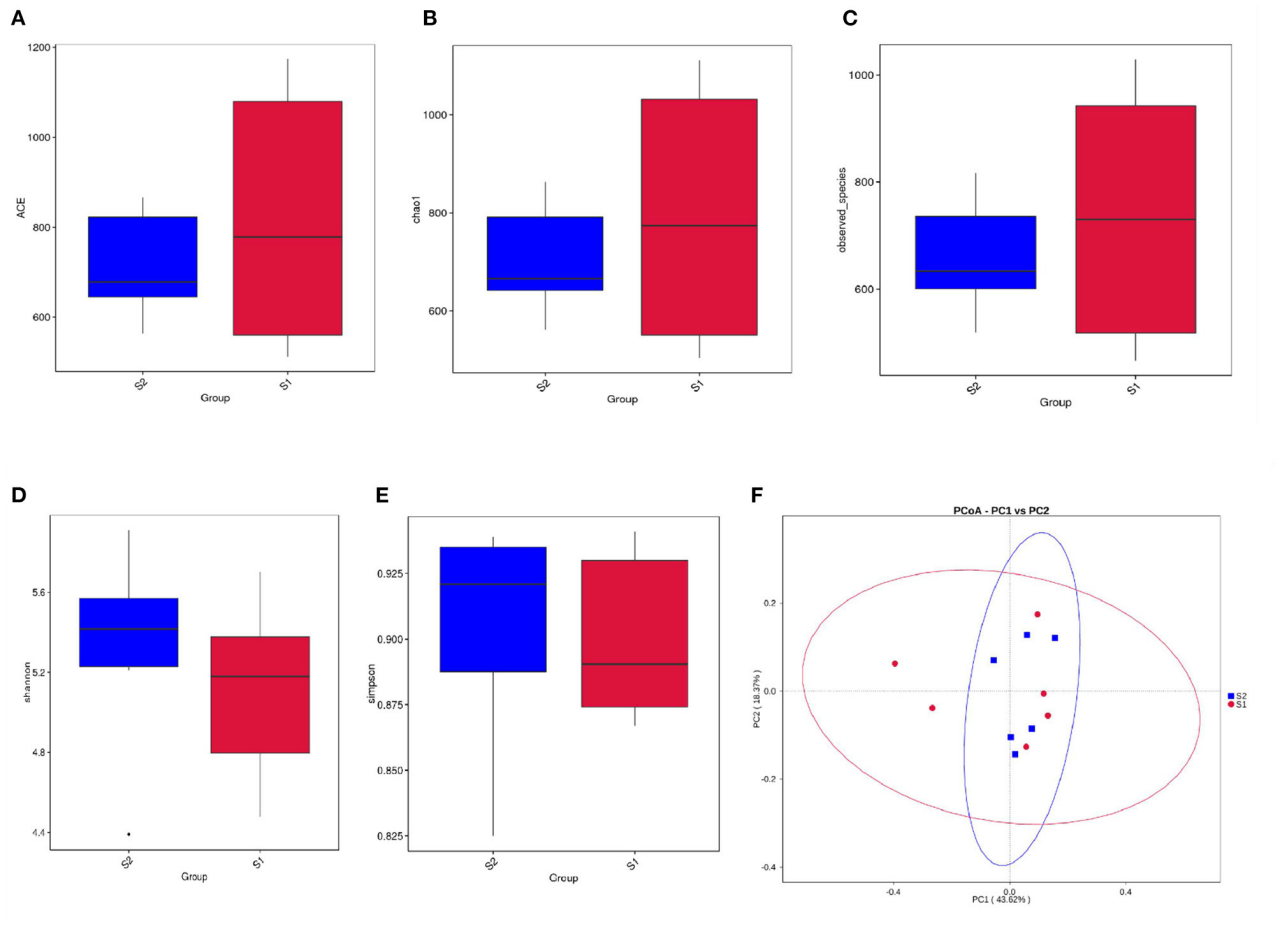
Effects of FWBPs administration on the alpha and beta diversity of the rumen microbiota in lambs. Analysis of the alpha and beta diversity *via* **(A)** ACE index, **(B)** Chao1 index, **(C)** Observed_species, **(D)** Shannon index, **(E)** Simpson index, and **(F)** principal coordinate analysis (PCoA) of weighted UniFrac distances, respectively. S1, the milk replacer (*n* = 6); S2, supplemented with 2‰ fermented wheat bran polysaccharides in the milk replacer (*n* = 6).

A total of 1,066,361 raw tags were generated across all 12 samples, with an average of 88,863 ± 3,876 raw tags per sample. After size filtering, quality control, and chimera removal, a total of 1,055,803 effective tags were obtained, and each ruminal digesta sample produced 87,983 ± 3,764 effective tags. For alpha diversity, there was no difference between the control and FWBPs groups regarding ACE, Chao1, Shannon, Observed_species, and Simpson indices (Figures 1A–E). The beta diversity analysis (PCoA based on weighted UniFrac distances) revealed no differences between the control and FWBPs groups (Figure 1F).

At the phylum level, Bacteroidetes, Firmicutes, and Proteobacteria were the three predominant phyla and accounted for more than 83% of rumen bacteria in lambs (Table 7). However, FWBPs treatment did not have an effect on the relative abundance of any phylum.

**Table 7:** Effects of FWBPs on the ruminal microbiota of early-weaned lambs (phylum level) %.

**Items**	**CON**	**FWBPs**	**SEM**	***p*-value**
Bacteroidota	39.66	38.9	3.495	1.00
Proteobacteria	22.76	24.60	3.101	0.82
Firmicutes	20.67	25.25	2.450	0.59
Actinobacteriota	8.00	1.34	2.296	0.39
Cyanobacteria	2.66	1.28	1.180	0.39
unidentified_Bacteria	2.56	4.63	0.753	0.24

*The relative abundance ≥1% for at least one group is presented.*

*CON, the milk replacer (n = 6); FWBPs, supplemented with 2‰ fermented wheat bran polysaccharides in the milk replacer (n = 6).*

*SEM, standard error of the mean*.

The relative abundances of the 35 most abundant genera are presented in Table 8. Lambs in the FWBPs group had a higher relative abundance of the *Lachnospiraceae- _NK3A20_group* and *Solobacterium* (*p* < 0.05) compared to lambs in the control group. Moreover, the relative abundance of *NK4A214_group, Megasphaera*, and *Treponema* had an increased trend (0.05 ≤ *p* < 0.10) in the FWBPs group.

**Table 8:** Effects of FWBPs on the ruminal microbiota of early-weaned lambs (genus level) %.

**Items**	**CON**	**FWBPs**	**SEM**	***p*-value**
*Prevotella*	20.52	22.82	2.479	0.785
*Succinivibrionaceae_UCG-001*	20.32	23.11	3.346	0.804
*Bifidobacterium*	6.42	0.17	2.145	0.119
*Lactobacillus*	4.92	1.62	1.592	0.362
*unidentified_Chloroplast*	2.43	0.76	1.208	0.656
*Dialister*	5.10	5.00	0.977	0.975
*Streptococcus*	1.18	0.55	0.506	0.691
*Sharpea*	0.96	0.25	0.354	0.376
*Prevotellaceae_YAB2003_group*	0.79	1.05	0.382	0.840
*NK4A214_group*	0.47	1.38	0.298	0.068
*Olsenella*	1.19	0.84	0.258	0.665
*Acidaminococcus*	0.65	1.00	0.193	0.519
*unidentified_Mitochondria*	0.44	0.02	0.219	0.499
*Prevotellaceae_UCG-001*	1.17	1.11	0.200	0.924
*Bacteroides*	1.11	0.77	0.171	0.492
*Succiniclasticum*	0.69	0.64	0.166	0.938
*Alloprevotella*	0.48	0.62	0.129	0.736
*Rikenellaceae_RC9_gut_group*	0.47	0.39	0.101	0.823
*Lachnospiraceae_NK4A136_group*	0.13	0.47	0.116	0.104
*Syntrophococcus*	0.31	0.60	0.102	0.135
*Megasphaera*	0.27	0.69	0.129	0.051
*Haemophilus*	0.10	0.48	0.134	0.149
*Neisseria*	0.25	0.39	0.132	0.749
*Ruminococcus*	0.37	0.33	0.080	0.873
*Escherichia-Shigella*	0.21	0.04	0.085	0.375
*Lachnospiraceae_NK3A20_group*	0.30	0.60	0.072	0.006
*Selenomonas*	0.37	0.52	0.064	0.288
*Fibrobacter*	0.29	0.26	0.075	0.912
*Dubosiella*	0.05	0.23	0.075	0.242
*Veillonella*	0.24	0.24	0.090	0.982
*Methanobrevibacter*	0.32	0.10	0.074	0.106
*Treponema*	0.04	0.21	0.057	0.075
*Solobacterium*	0.12	0.29	0.050	0.040
*Elusimicrobium*	0.12	0.03	0.051	0.544
*Enterorhabdus*	0.16	0.11	0.050	0.722

*The relative abundances of the 35 most abundant phyla are presented.*

*CON, the milk replacer (n = 6); FWBPs, supplemented with 2‰ fermented wheat bran polysaccharides in the milk replacer (n = 6).*

*SEM, standard error of the mean*.

**Figure 2:**
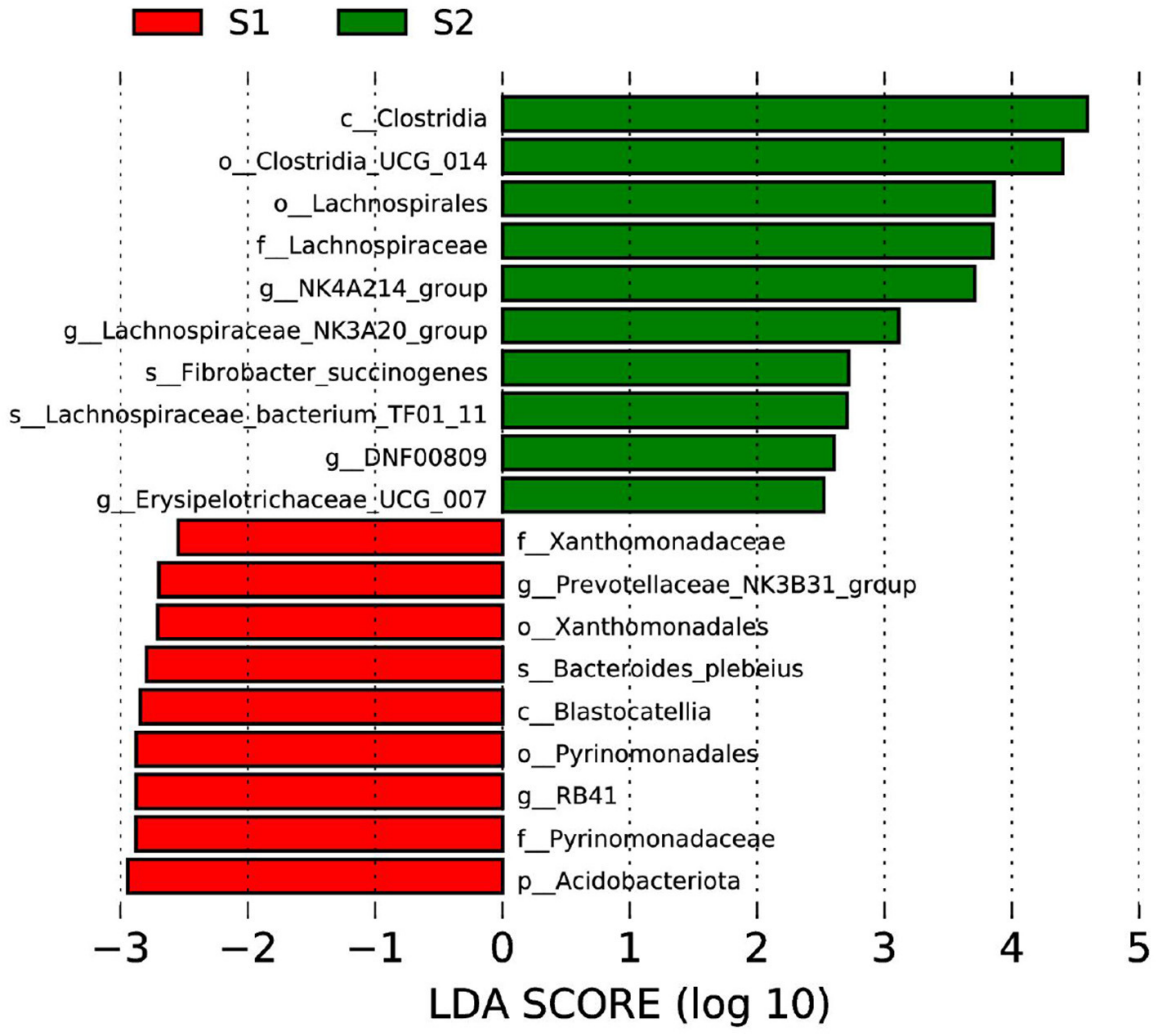
Bacterial taxa differences between control and FWBPs groups. Linear discriminant analysis (LDA) coupled with effect size (LEfSe) analysis was performed to identify the bacterial taxa differentially represented in control and FWBPs groups at different taxonomy levels. S1, the milk replacer (*n* = 6); S2, supplemented with 2‰ fermented wheat bran polysaccharides in the milk replacer (*n* = 6).

The LEfSe approach was performed to identify the specific bacterial taxa that differed between control and FWBPs groups at different taxonomic levels (Figure 2). Lambs in the FWBPs group enriched Clostridia at the class level, Clostridia_UCG_014 and Lachnospirales at the order level, Lachnospiraceae at the family level, *NK4A214_group, Lachnospiraceae_NK3A20_group, DNF00809*, and *Erysipelotrichaceae_UCG_007* at the genus level, and *Fibrobacter_succinogenes* and *Lachnospiraceae_bacterium_TF01_11* at the species level. Lambs in the control group enriched Acidobacteriota at the phylum level, Blastocatellia at the class level, Xanthomonadales and Pyrinomonadales at the order level, Xanthomonadaceae and Pyrinomonadaceae at the family level, *Prevotellaceae_NK3B31_group* and *RB41* at the genus level, and *Bacteroides_plebeius* at the species level.

### Correlation Between Altered Bacterial Composition and Rumen Fermentation Parameters

**Figure 3:**
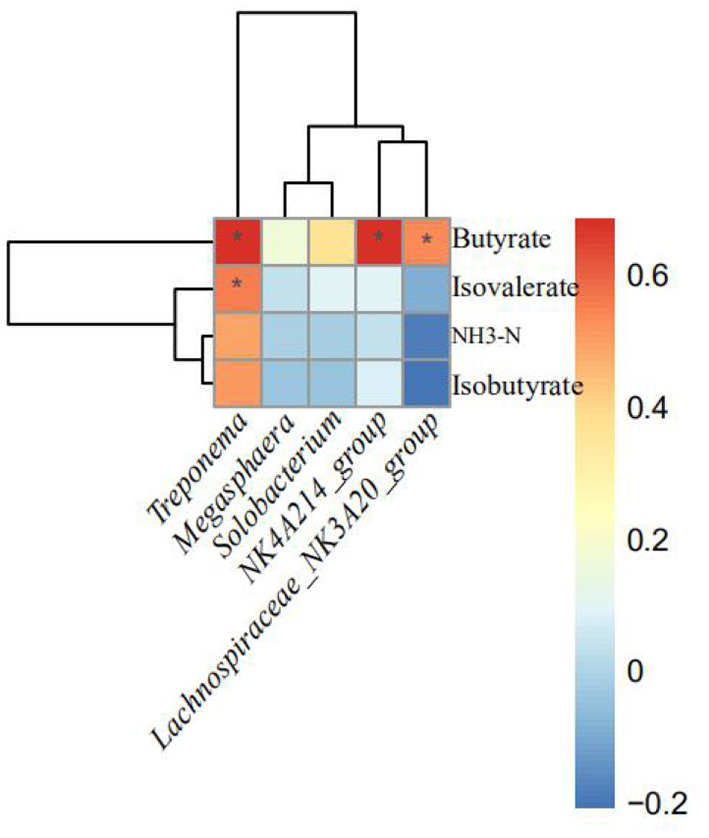
Correlation between the altered rumen bacteria (genus) and rumen fermentation indices. Significant differences are indicated as *p < 0.1. NH_3_-N, ammonia nitrogen.

As shown in Figure 3, the relative abundances of *NK4A214_group, Treponema*, and *Lachnospiraceae_NK3A20_group* were positively correlated with butyrate proportion (*p* < 0.10). In addition, the relative abundances of *Treponema* were positively correlated with isovalerate proportion.

## Discussion

Microbiota colonization is crucial for the development of the rumen and can have long-term effects on the performance of young ruminants (20). FWBPs are indigestible but fermentable natural biomacromolecules with biological roles, especially bacteria-modulating capacity (21, 22). In the current study, we used a suckling lamb model to study the promoting effects of FWBPs on growth performance and rumen development by manipulating rumen bacterial community. A previous study showed that that the addition of *boulardii yeast* cell wall polysaccharides in MR increased the ADG and feed conversion rate of early-weaned lambs (23). Consistent with the previous results, we demonstrated that FWBPs administration increased the ADG, final BW, and carcass BW and decreased the F:G ratio in the present study. The increased ADG, final BW, and carcass BW may be attributable to the infusion of FWBPs, which may improve rumen development and offer more energy for the lamb's growth.

The process of rumen development involves improvement of rumen mass as well as the growth of ruminal epithelium (24). In this study, the rumen weight of the lambs was found to be not influenced by FWBPs. However, the rumen weight in the FWBPs group increased by 9.12% compared with the control group, which indicated that FWBPs could improve rumen mass. The ruminal epithelium consists of rumen papillae, muscular layer, and epithelial cell and performs many important functions, including absorption, transportation, metabolism, and protection (25). The proliferation of the rumen epithelium cell promotes the growth of papillae length and width and increases the thickness of the muscular layer (26).

In this study, infusion with FWBPs enhanced the ruminal papillae width and epithelial cell thickness but had no effect on ruminal papillae length or muscle layer thickness. It has been reported that supplementation of alfalfa hay (rich in pectins) improved muscular layer thickness but had no effects on the height and width of ruminal papillae and epithelial cell thickness of Baluchi lambs (27). The difference between our results and previous reports might reflect that different types of plant polysaccharides have different effects on rumen development, which need further study. The VFAs can stimulate the growth of the ruminal epithelium, with the effect of butyrate being the most prominent, followed by propionate and acetate. Butyrate is known to reduce ruminal epithelial cell apoptosis, accelerate ruminal epithelium growth and maturation, and promote rumen development (28, 29). The present results showed that infusion with FWBPs increased the butyrate proportion in rumen contents of lambs. Wang et al. (9) found that FWBPs administration did not affect the butyrate proportion in rumen, which showed inconsistency with our results. The difference between our findings and previous reports might reflect that the butyrate response to FWBPs addition was animal growth stage as well as physiology dependence. The accumulation of ruminal VFAs can cause low ruminal pH. Coincidentally, a lower ruminal pH was observed in the FWBPs group, which was possibly due to the addition of FWBPs to MR, promoting the fermentation to produce more butyrate. Several studies have been shown that butyrate supplemented in MR stimulated the ruminal epithelium growth in newborn lambs and calves (30, 31). Therefore, we speculated that FWBPs are ingested into rumen and fermented to butyrate, which could stimulate the growth of the ruminal epithelium and promote the development of the rumen. In addition, FWBPs treatment decreased the isobutyrate and isovalerate proportion in rumen contents of lambs. Isobutyrate and isovalerate, which belong to branched-chain acids, are naturally derived from the fermentation of protein (32). The process of fermentation also produced potentially hazardous chemicals (amines, phenols, cresols, and hydrogen sulfide) (32). As a result, the decrease of isobutyrate and isovalerate may be helpful to maintain rumen health due to lower potentially toxic compound concentrations. Ruminal NH_3_-N is the most important source for microbial protein synthesis (33). Results of the present study suggested that the concentration of NH_3_-N was decreased in response to the infusion of FWBPs. Lower NH_3_-N concentrations in the FWBPs group could be attributed to increased microbial protein synthesis, resulting in more effective ruminal NH_3_-N transport into the body which has been confirmed by increased serum total protein concentrations of lambs fed with FWBPs. The findings are consistent with those of the previous study, which found that treatment with FWBPs lowered ruminal NH_3_-N concentrations and increased serum total protein concentrations (9).

The rumen microbiota is responsible for converting normally indigestible feedstuffs into VFAs and microbial protein in ruminants (34, 35). Herein, we evaluate the effects of FWBPs supplementation to MR on the structure and composition of ruminal bacteria by high-throughput sequencing of the 16S rRNA gene V4 region and the relationship between altered rumen bacteria (genus) and rumen fermentation parameters by Spearman's correlation analysis. In this study, there was no significant difference between the control and FWBPs groups regarding the alpha and beta diversity of ruminal bacteria. Chen et al. found that supplementation with Chinese medicine polysaccharides had no significant effect on the alpha and beta diversity of rumen bacteria in the lambs, which validated our obtained results (36). Another study by Yang et al. (37) found that alfalfa intervention had no effect on the alpha diversity of ruminal bacteria in Hu lambs (age 66 days). The current study showed that Bacteroidetes, Firmicutes, and Proteobacteria were the three core phyla, which is consistent with the results by Zhang et al. (35). Feeding with FWBPs had no effect on the structure and composition of ruminal bacteria at the phylum level. However, administration of FWBPs increased the relative abundance of *Lachnospiraceae_NK3A20_group, Solobacterium, NK4A214_group, Megasphaera*, and *Treponema* at the genus level. The bacterial genera *Lachnospiraceae_NK3A20_group, NK4A214_group*, and *Treponema*, belonging to fibrolytic bacteria, are closely related to the VFAs production (38–40). Furthermore, the relative abundance of *Lachnospiraceae_NK3A20_group, NK4A214_group*, and *Treponema* and butyrate proportion was found to be positively correlated. Similar results were observed by Huang et al., where the *Lachnospiraceae NK3A20 group* not only promotes rumen development by increasing butyrate production but also directly affects rumen development (41). *Solobacterium* is a member of the family Erysipelotrichaceae of the phylum Firmicutes, which is involved in the digestion of protein and energy production (42). *Megasphaera*, as rumen probiotics, can convert lactate to acetate, propionate, and butyrate, which could help in energy balance and animal productivity (43, 44). Furthermore, the LEfSe analysis revealed that lambs in the FWBPs group enriched differential OTUs; most of these OTUs belong to the class Clostridia (Clostridia_UCG_014, Lachnospirales, Lachnospiraceae, *NK4A214_group, Lachnospiraceae_NK3A20_group*, and *Lachnospiraceae_bacterium_TF01_11*). The class Clostridia includes many polysaccharolytic bacteria that contribute to the production of VFAs in the gut (45). In addition, *Fibrobacter_succinogenes* is also enriched in lambs fed with FWBPs. *Fibrobacter_succinogenes* is a cellulolytic rumen bacteria that is known to utilize NH_3_-N as their sole source of nitrogen (46, 47). Based on the present results and references, we speculated that FWBPs supplementation with MR stimulated the proliferation of fibrolytic bacteria including *Lachnospiraceae_NK3A20_group, NK4A214_group, Treponema*, and *Fibrobacter_succinogenes* and promoted the presence of VFAs producers *Megasphaera*, which could increase VFAs and microbial protein production, accelerate development of the rumen, and then improve growth performance in the early weaned lambs.

## Conclusion

In summary, MR with FWBPs increased ADG, final BW, carcass BW, serum total protein concentrations, ruminal papillae width, epithelial cell thickness, and ruminal butyrate proportion, while it decreased the F:G ratio, the concentration of NH_3_-N, and the proportion of isobutyrate and isovalerate. Furthermore, the relative abundance of *Lachnospiraceae_NK3A20_group, Solobacterium, NK4A214_group, Megasphaera*, and *Treponema* was enhanced by FWBPs treatment. These findings suggested that FWBPs supplementation to MR on suckling lambs altered rumen bacterial community, promoted rumen development, and improved growth performance.

## Data Availability Statement

The datasets analyzed during the current study are available in the SRA NCBI repository under the Bioproject accession number (PRJNA791141).

## Ethics Statement

The current study approval for experimental protocols on animals was provided by the Animal Care and Use Committee of Inner Mongolia Agricultural University [(2020)069].

## Author Contributions

YW, XA, and JQ conceived and designed the experiment. WW, ZC, and YY collected the raw materials and performed the experiment. WW analyzed the data and interpreted the results. WW and YW wrote the manuscript. All authors contributed to the article and approved the submitted version.

## Funding

This research was supported by the Major Science and Technology Program of Inner Mongolia Autonomous Region (2021ZD0023-3, 2021ZD0024-4, 2020ZD0004), Key Technology Project of Inner Mongolia Autonomous Region (2020GG0030), and Natural Science Foundation of Inner Mongolia Autonomous Region (2020MS03041).

## Conflict of Interest

The authors declare that the research was conducted in the absence of any commercial or financial relationships that could be construed as a potential conflict of interest.

## Publisher's Note

All claims expressed in this article are solely those of the authors and do not necessarily represent those of their affiliated organizations, or those of the publisher, the editors and the reviewers. Any product that may be evaluated in this article, or claim that may be made by its manufacturer, is not guaranteed or endorsed by the publisher.
